# Projection Patterns of Corticofugal Neurons Associated with Vibrissa Movement

**DOI:** 10.1523/ENEURO.0190-18.2018

**Published:** 2018-10-19

**Authors:** Ken-Ichi Shibata, Takuma Tanaka, Hiroyuki Hioki, Takahiro Furuta

**Affiliations:** 1 Department of Morphological Brain Science, Graduate School of Medicine, Kyoto University, Kyoto 606-8501, Japan; 2 Department of Oral Anatomy and Neurobiology, Graduate School of Dentistry, Osaka University, Suita 565-0871, Japan; 3 Faculty of Data Science, Shiga University, Hikone 522-8522, Japan; 4 Department of Cell Biology and Neuroscience, Graduate School of Medicine, Juntendo University, Tokyo 113-8421, Japan

**Keywords:** intratelencephalic neuron, pyramidal tract neuron, single cell electroporation, whisker

## Abstract

Rodents actively whisk their vibrissae, which, when they come in contact with surrounding objects, enables rodents to gather spatial information about the environment. Cortical motor command of whisking is crucial for the control of vibrissa movement. Using awake and head-fixed rats, we investigated the correlations between axonal projection patterns and firing properties in identified layer 5 neurons in the motor cortex, which are associated with vibrissa movement. We found that cortical neurons that sent axons to the brainstem fired preferentially during large-amplitude vibrissa movements and that corticocallosal neurons exhibited a high firing rate during small vibrissa movements or during a quiet state. The differences between these two corticofugal circuits may be related to the mechanisms of motor-associated information processing.

## Significance Statement

The contents of cortical motor commands and the pathways that they take are two important questions in neuroscience. Previous studies in rodents reported that motor cortex neurons exhibit variable activity patterns during whisking behavior. We combined physiologic and morphologic analyses of single neurons to provide more detail for understanding the neural circuit related to motor control.

## Introduction

Rodents use vibrissae, which are long hairs on the sides of their faces, as tactile sensors that help them gather spatial information about the environment (Diamond et al., 2008; Kleinfeld and Deschênes, 2011). Specifically, rodents can obtain tactile descriptions of texture, shape, and position by actively moving their vibrissae to touch objects (Welker, 1964; Mitchinson et al., 2011). In a typical vibrissal movement pattern, which is called “whisking,” rodents repeatedly sweep their vibrissae back and forth such that they contact any objects located in the space surrounding the head of the animal. During exploratory behavior, the speed and amplitude of whisking are precisely adjusted by the vibrissal motor system depending on the situation and chosen behavioral strategies (Towal and Hartmann, 2006; Mitchinson et al., 2007; Grant et al., 2009; Arkley et al., 2014). Stimulation of the vibrissal primary motor cortex (vM1) has been found to evoke vibrissa movement (Donoghue and Wise, 1982), and vM1 neurons modulate whisking parameters such as frequency and amplitude (Hill et al., 2011; Gerdjikov et al., 2013). Cortical motor commands are considered to play an important role in whisking behavior (Petersen, 2014). The whisking central pattern generator (CPG) in the brainstem controls the facial nuclei (FNs; Moore et al., 2013; Takatoh et al., 2013), which directly drive vibrissa movement (Herfst and Brecht, 2008). The CPG receives cortical input from layer 5 of the vM1 (Alloway et al., 2010; Petersen, 2014). Layer 5 pyramidal neurons of the vM1 are known to include two morphologic groups, intratelencephalic (IT) neurons and pyramidal tract (PT) neurons (Shepherd, 2013; Harris and Shepherd, 2015; Kawaguchi, 2017). IT neurons project axons bilaterally only within the telencephalon (mainly the cerebral cortex and striatum; Veinante and Deschênes, 2003; Alloway et al., 2009), and PT neurons send axon collaterals ipsilaterally to the cerebral cortex and to subcortical structures on the way to the brainstem or spinal cord (Deschênes et al., 1994; Alloway et al., 2008; Kress et al., 2013). In the vibrissa system, these architectures imply that PT neurons provide motor commands mainly for the ipsilateral cerebral cortex and subcortical structures while IT neurons send motor information only to the bilateral telencephalon. Thus, motor commands conveyed by PT neurons appear to be different from motor-associated information encoded by IT neurons.

Here, we investigated correlations between axonal projection patterns of layer 5 neurons in the vM1 and neuronal firing properties associated with vibrissa movement. Such correlations may reveal how corticofugal circuits contribute to vibrissal motor control and provide a platform for the integration of vibrissa motor information with other signals, such as sensory inputs.

## Materials and Methods

### Experimental design

We used male Long-Evans rats (weighing 250–350 g) for studies involving intracortical microstimulation (*n* = 6), electrophysiology and anatomy (*n* = 40). Approximately 95 coronal brain slices and 220 sagittal slices per animal were obtained in the anatomic study. All procedures were conducted in accordance with the animal care guidelines recommended by the Institute of Laboratory Animals, Graduate School of Medicine, Kyoto University (approved number: MedKyo16573). All efforts were made to minimize the suffering and number of animals used in this study.

### Intracortical microstimulation study

The three rats were anesthetized via an intraperitoneal injection of chloral hydrate (35 mg/100 g body weight), and the other three rats were under ketamine (75 mg/kg; xylazine, 5 mg/kg) anesthesia. They were fixed in place in a stereotaxic instrument. A craniotomy was performed over the frontal cortex. A single 250-µm diameter parylene-coated tungsten electrode with an impedance of 2 MΩ (A-M Systems) was successively lowered at a depth 1500 µm below the pia surface to stimulate various locations within the cortex. We used two microstimulation paradigms to locate areas that led to vibrissal motion. The bipolar current pulses (cathodal first) were delivered at 60 Hz with a duration of 200 µs (20–100 µA; Haiss and Schwarz, 2005). The monophasic cathodal pulses of 100 µA with a duration of 200 µs each were delivered thorough the microelectrode at 2-ms intervals (Donoghue and Wise, 1982). Visual inspection under a microscope confirmed that the vibrissae moved in response to stimulation.

### Surgery

**Figure 1.**
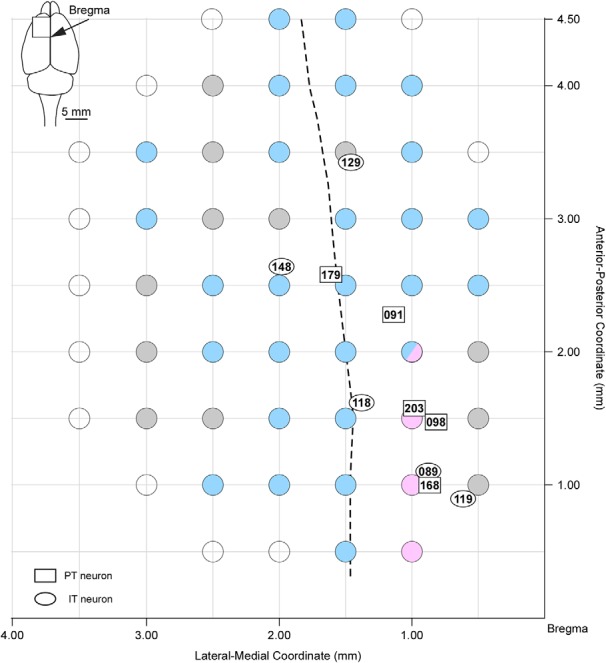
Vibrissa movement map of responses to intracortical microstimulation in the frontal cortex. Microstimulations evoked protoractions (magenta filled circles), retractions (cyan filled circles), and vibrations of small amplitudes without any dominance of protraction or retraction (gray filled circles). Microstimulations in a location (2 mm rostral, 1 mm lateral to the bregma) induced both protractions (one rat) and retractions (two rats). Open circles indicate places where the microstimulations did not cause any vibrissa movement. Rectangles and ovals indicate the locations of cell bodies of PT and IT neurons, respectively. Numbers within symbols denote the neuron number. Dashed line shows the boundary between the AGm and AGl at a depth of layer 5.

Rats were briefly handled. After the rats were anesthetized via an intraperitoneal injection of chloral hydrate (35 mg/100 g body weight), a lightweight head attachment (Narishige Co.) was surgically attached to the skull, and a reference electrode was implanted in the occipital bone. Two small portions of the left side of the skull were removed: one was located 1.5 mm anterior and 0.75 mm lateral from bregma, and the other was located 2.5 mm anterior and 1.5 mm lateral from bregma (Kleinfeld et al., 2002; Haiss and Schwarz, 2005). Those areas corresponded to the left hemisphere vM1 (Fig. 1). We trimmed all vibrissae on the right side, except the second vibrissa in row C (C2). Retrimming occurred every third day.

### Videography

**Figure 2.**
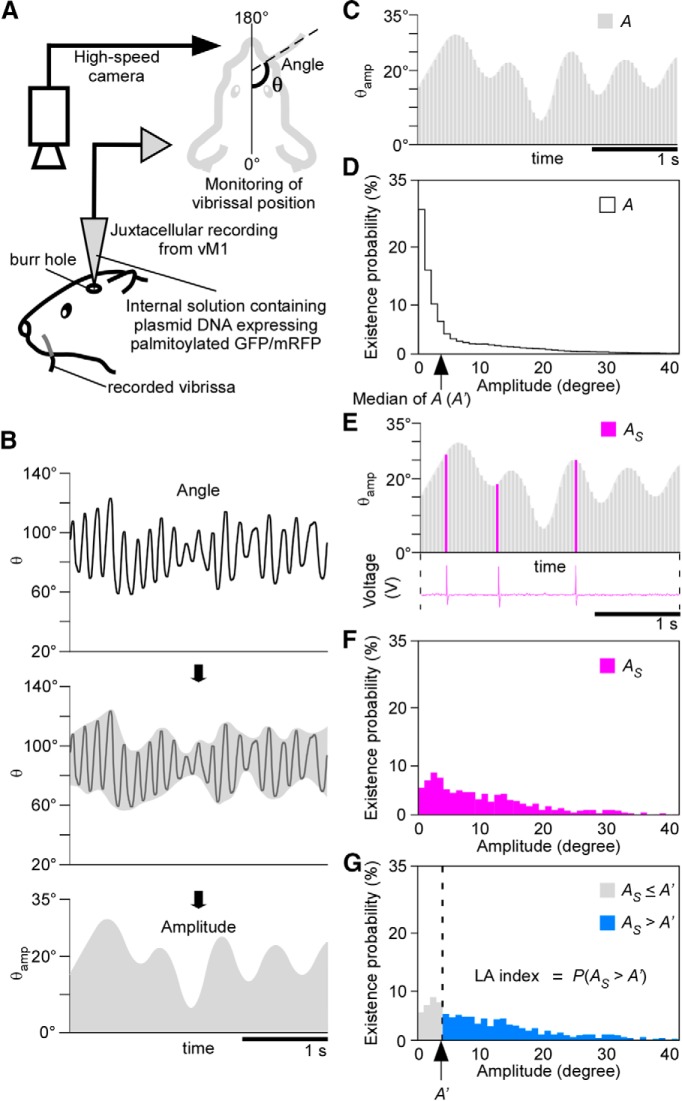
The LA index represents the preference of each unit for large whisking. ***A***, Schematic diagram describing vibrissal position monitoring via a high-speed camera and juxtacellular recording from the vM1 on the side contralateral to the recorded vibrissa. ***B***, Whisk amplitude (*θ_amp_*) was calculated using the wavelet transform and the Hilbert transform. ***C***, We continuously measured whisk amplitude (*A*) while recording neuronal activity. ***D***, Histogram showing the distribution of the existence probabilities of *A*. The median of *A* (*A’*) in this neuron was 4° (arrow). ***E***, *A_S_* was defined as the whisk amplitude during instances of neuronal spiking. ***F***, Histogram showing the distribution of the existence probabilities of *A_S_*. ***G*,** The LA index was defined as *P*(*A_S_* > *A’*).

We monitored the position of the right C2 vibrissa in the head-fixed rats using a high-speed camera (CV-035M, Keyence) at 200 Hz (Fig. 2*A*). Planar images (512 × 480 pixel) were acquired at a spatial resolution of 100 µm with an infrared light-emitting diode (850 nm) backlight. An image sensor (CV-5500, Keyence) received data from the high-speed camera and calculated the angle (*θ*) formed between the anterior-posterior axis of the rat and the center of the vibrissa. We acquired vibrissa movement as the angle *θ*(t), where t was the discrete time, on a computer. The time delay (21.8 ms), which was caused by the image processing, was compensated in the data analysis.

### Behavioral training

After recovering from surgery, the rats were deprived of drinking water in their home cages. The rats were trained to perform a voluntary whisking task for 2 h in an operant system (O’hara & Co.). Training sessions were scheduled two times per day for 5 d. We used a personal computer (Dell) with LabVIEW software (National Instruments) to control the training apparatuses. The rats were monitored using an infrared camera. A 10-ms 6-kHz tone indicated the start of each trial. If a rat continued moving its vibrissae with a frequency >3 Hz and amplitude >3° for 1 s, he received a drop of water from a spout connected to a syringe pump. The intervals between trials ranged from 3000 to 4000 ms.

### Decomposition of vibrissa movement

To minimize errors associated with videography, the angle (*θ*) was corrected to an averaged angle. The averaged angle was the arithmetic mean of the three middle of nine continuous values of the angle occurring from *θ*(t–4) to *θ*(t + 4). We obtained the high-frequency components of the time series of the averaged angle by setting the coefficients of the levels of decomposition corresponding to ≥64 samples in the biorthogonal B-spline wavelet transform of order (3, 9) to zero. We acquired the amplitude signal *θ*_amp_(t) from the absolute values of the high-frequency component using the Hilbert transform (Fig. 2*B*).

### Electrophysiological recording and electroporation

We inserted either the palmitoylation site-attached green fluorescent protein (palGFP; Furuta et al., 2001) sequence or the monomeric red fluorescent protein (pal-mRFP; Nishino et al., 2008) sequence into the EcoRI/NotI sites of pCAG-EN (Addgene plasmid 11160), resulting in pCAG-palGFP or pCAG-pal-mRFP. The glass electrode (tip resistance, 8 MΩ; tip diameter, 1 µm) was filled with a standard internal solution (140 mM K-gluconate, 5 mM KCL, 1 mM MgCl_2_, 2 mM EGTA, 5 mM HEPES, and 2 mM MgATP; pH 7.2) and 500 ng/µl plasmid, along with pCAG-palGFP or pCAG-pal-mRFP. The electrode was attached to a pipette holder (MEH2SW, World Precision Instruments) and a fine micromanipulator (DMA-1511, Narishige Co.) on a stereotaxic frame (SR-8N-S, Narishige Co.) and was lowered vertically to record layer 5 pyramidal cells (depth: 1000–2000 µm below the pia) in the two areas of the left vM1 where the skull had already been removed. Extracellular signals were amplified (IR-283, Cygnus Tech), bandpass filtered (100 Hz to 2 kHz, 440 instrumentation amplifier, Brownlee Precision), sampled at 10 kHz, and stored on a hard disk for off-line analysis. After completing electrophysiological recording, some recorded neurons (one to two neurons in an animal) received plasmid electroporation. The electrode was disconnected from the amplifier and connected to a stimulus isolator (SS-202J and SEN-301, Nihon Kohden). We applied a cathodal voltage pulse train (–10 V, 50 pulses at 50 Hz) to inject the plasmid into the recorded cell (Oyama et al., 2013). The plasmid injections were performed with enough distances (500-µm anterior-posterior coordinate, 1000-µm lateral-medial coordinate) between places of the injections. This enabled us to identify each individual labeled neuron in the tissue sections and associate them with the appropriate electrophysiological records.

### Fixation of the brain

The rats were sacrificed 10 d after the plasmid injection. The rats were anesthetized with chloral hydrate (70 mg/100 g) and perfused transcardially with 200 ml of 5 mM sodium phosphate-buffered 0.9% saline (PBS; pH 7.4), followed by 200 ml of 3.7% (w/v) formaldehyde in 0.1 M sodium phosphate buffer (PB; pH 7.4). The brains were removed and postfixed for 2 h at room temperature with the same fixative. After the brains were cryoprotected with 30% (w/v) sucrose in PBS at room temperature overnight, the front portions of the brains (from the frontal pole of the cerebrum to 0.96 mm posterior to bregma) were sliced into 60-µm-thick coronal sections and the rear portions (from 0.96 mm posterior to bregma to the brainstem) were sliced into 60-µm-thick sagittal sections using a freezing microtome. Locations were determined using a stereotaxic atlas (Paxinos and Watson, 2007). Sections were serially collected in PB.

### Histologic staining

All sections were incubated overnight with 0.3 µg/ml affinity-purified rabbit anti-GFP antibody (Nakamura et al., 2008) or 0.15 µg/ml affinity-purified rabbit anti-mRFP antibody (Hioki et al., 2010) in PBS containing 0.3% Triton X-100, 0.12% lambda-carrageenan, 0.02% sodium azide, and 1% donkey serum (PBS-XCD). After a rinse with PBS containing 0.3% Triton X-100 (PBS-X), the sections were incubated for 1 h with 10 µg/ml biotinylated goat anti-rabbit IgG antibody (BA-1000; Vector) and then for 1 h with avidin-biotinylated peroxidase complex (ABC; 1:100; Elite variety, Vector) in PBS-X. After a rinse in 0.1 M PB, we applied the biotinylated tyramine (BT)-glucose oxidase (GO) amplification method (Furuta et al., 2009) to the sagittal sections. The sagittal sections were incubated for 30 min in the BT-GO reaction mixture containing 1.37 µM BT, 3 µg/ml of GO (273 U/mg; Nacalai Tesque), 2 mg/ml β-D-glucose, and 1% BSA in 0.1 M PB, followed by a wash with PBS-X. Subsequently, the sagittal sections were again incubated for 1 h with ABC-Elite in PBS-X. The peroxidase in all sections was visualized using 0.025% diaminobenzidine-4HCl (DAB; Dojindo), 0.625% nickel, and 0.00015% H_2_O_2_ in 50 mM Tris-HCl (pH 7.6). All incubations were performed at room temperature. All stained sections were serially mounted onto gelatin-coated glass slides and dried overnight. The sections were cleared in ethanol and xylene and then coverslipped with organic mounting medium MX (Matsunami). After reconstruction of palGFP- or pal-mRFP-labeled neurons, the sections were counterstained for Nissl with 1% neutral red to identify cytoarchitecture. The cytoarchitecture was determined based on a stereotaxic atlas (Zilles, 1985; Paxinos and Watson, 2007) and previous reports (Zilles et al., 1980; Kubota et al., 2007; Smith and Alloway, 2013).

### Reconstruction and morphologic analysis of single vM1 neurons

Whole coronal or sagittal sections were automatically captured as large color images using a TOCO digital slide scanner (CLARO) with a 10× objective lens [EC Plan-Neofluar; Zeiss; numerical aperture (NA) = 0.30]. The obtained images had a spatial resolution of 1.038 × 1.038 µm, and the axon fibers were traced and digitized using Canvas 12 software (ACD System International Inc.). We reconstructed the axon fibers as a collection in a two-dimensional plane using computer software (Ohno et al., 2012). Axon density in each targeted brain area was semi-quantitatively evaluated (Tables 1, 2). The cell body and dendrites were three-dimensionally reconstructed using Neurolucida computer-assisted neuron tracing system 11 (MBF Bioscience). The reconstructed dendrites were quantitatively analyzed via Sholl analysis (Sholl, 1953) with Neurolucida-associated software NeuroExplorer (MBF). Data were analyzed per 25-µm concentric circles.

**Table 1.** Summary of the distribution of labeled axon fibers ipsilateral to the cell body

			Cerebral cortex					Striatum	Diencephalon					Midbrain				Pons		Medulla oblongata			
Neuron number	LA index	Projection type							Thalamus														
			Cg	AGm	AGl	FL	S1BF	CPu	AV	POm	STN	ZI	Hb	APT	RN	SC	PAG/mRt	PN	PnO/PnC	Gi	IO	IRt	MV
148	0.2409	IT	−	++	++*	++	++	+++	−	−	−	−	−	−	−	−	−	−	−	−	−	−	−
089	0.3870	IT	++*	++	++	−	−	−	−	−	−	−	−	−	−	−	−	−	−	−	−	−	−
168	0.4070	PT	−	++*	−	−	−	+	−	+	+	++	−	+	−	+	++	+++	−	−	−	−	−
129	0.4665	IT	−	++*	++	−	++	−	−	−	−	−	−	−	−	−	−	−	−	−	−	−	−
118	0.4825	IT	+	++*	++	−	−	++	−	−	−	−	−	−	−	−	−	−	−	−	−	−	−
119	0.5147	IT	++*	−	−	−	−	+++	−	−	−	−	−	−	−	−	−	−	−	−	−	−	−
179	0.5149	PT	−	+++	+++*	+	−	+	−	++	+	+++	−	+	−	++	++	+++	++	++	−	+++	++
203	0.5987	PT	+	++*	−	−	−	++	−	++	+	++	−	+	−	++	+++	++	−	+	+	−	−
091	0.6875	PT	−	++*	+	−	−	++	−	−	+	++	−	+	+	+	+++	++	+++	++	+	++	++
098	0.7434	PT	++	++*	−	+	−	++	+	−	+	++	+	−	−	+	+++	++	++	+	−	−	−

Axon density was semi-quantitatively evaluated and classified according to the following standards: +++, two or more axonal clusters; ++, one cluster; +, no cluster but axon fibers present; −, no axon fibers. * indicates the location of the cell body. The vM1 was confirmed by intracortical microstimulation. The vM1 area straddled the Cg, as well as the medial and lateral agranular cortices (AG). AGl, lateral agranular cortex; AGm, medial agranular cortex; APT, anterior pretectal nucleus; AV, anteroventral thalamic nucleus; CPu, caudate putamen; FL, forelimb area; Gi, gigantocellular reticular nucleus; Hb, habenular nucleus; IO, inferior olive; IRt, intermediate reticular nucleus; LA index; large-amplitude index; MV, medial vestibular nucleus; mRt, mesencephalic reticular formation; PAG, periaqueductal gray; PN, pontine nuclei; PnC, caudal part of pontine reticular nucleus; PnO, oral part of pontine reticular nucleus; POm, posterior thalamic nuclei; RN, red nucleus; SC, superior colliculus; STN, subthalamic nucleus; S1BF, barrel field of primary somatosensory cortex; ZI, zona incerta.

**Table 2.** Summary of the distribution of labeled axon fibers contralateral to the cell body

			Cerebral cortex					Striatum	Diencephalon					Midbrain				Pons		Medulla oblongata			
Neuron number	LA index	Projection type							Thalamus														
			Cg	AGm	AGl	FL	S1BF	CPu	AV	POm	STN	ZI	Hb	APT	RN	SC	PAG/mRt	PN	PnO/PnC	Gi	IO	IRt	MV
148	0.2409	IT	−	+	++	−	−	+++	−	−	−	−	−	−	−	−	−	++	−	−	−	−	−
089	0.3870	IT	++	+	−	−	−	++	−	−	−	−	−	−	−	−	−	++	−	−	−	−	−
168	0.4070	PT	−	−	−	−	−	−	−	−	−	−	−	−	−	−	−	++	−	−	−	−	−
129	0.4665	IT	−	−	−	−	−	+++	−	−	−	−	−	−	−	−	−	−	−	−	−	−	−
118	0.4825	IT	−	+	−	−	−	++	−	−	−	−	−	−	−	−	−	−	−	−	−	−	−
119	0.5147	IT	++	−	−	−	−	++	−	−	−	−	−	−	−	−	−	−	−	−	−	−	−
179	0.5149	PT	−	−	−	−	−	−	−	−	−	−	−	−	−	−	−	−	++	+	−	++	++
203	0.5987	PT	−	−	−	−	−	−	−	−	−	−	−	−	−	−	−	−	−	−	+	−	−
091	0.6875	PT	−	−	−	−	−	−	−	−	−	−	−	+	+	−	+	++	++	+	−	++	++
098	0.7434	PT	−	−	−	−	−	−	−	−	−	−	−	−	−	−	−	+	−	−	−	−	−

Semi-quantitative evaluation of axon density. Criteria and abbreviations are the same as those used in Table 1.

### Statistics

We performed statistical analyses using the MATLAB Statistics toolbox. We used an *F* test to compare the distribution of the LA index of the recorded neurons (*n* = 203) with that of the shuffled data. We compared the LA indexes of the analyzed PT neurons and those of the IT neurons using the Wilcoxon rank-sum test. We also used the Wilcoxon rank-sum test to compare the number of dendritic intersections in the PT and IT neurons. The significance level was set at *p* < 0.05. Group data were expressed as mean ± SD.

## Results

Although previous studies have reported that intracortical microstimulation of the anterior and medial areas of the primary motor cortex (M1) evoke vibrissa movement, the exact location of the vM1 is controversial (Donoghue and Wise, 1982; Kleinfeld et al., 2002; Haiss and Schwarz, 2005). We used bipolar and monophasic stimulation procedures (see Methods) and observed vibrissa movement evoked by microstimulation. Because the penetrations of the electrical recordings in the present study were located within the area where vibrissa movement was elicited by microstimulation (Fig. 1), the recorded neurons was considered to be included in the vM1. Our data indicated that the vM1 area straddled the cingulate cortex (Cg) and the medial agranular cortex (AGm) and lateral agranular cortex (AGl) in agreement with the cortical maps of vibrissal movement of previous studies (Kleinfeld et al., 2002; Haiss and Schwarz, 2005). All the neurons described below were recorded in layer 5 of the vM1.

### Correlation between whisk amplitude and firing probability of vM1 neurons

**Figure 3.**
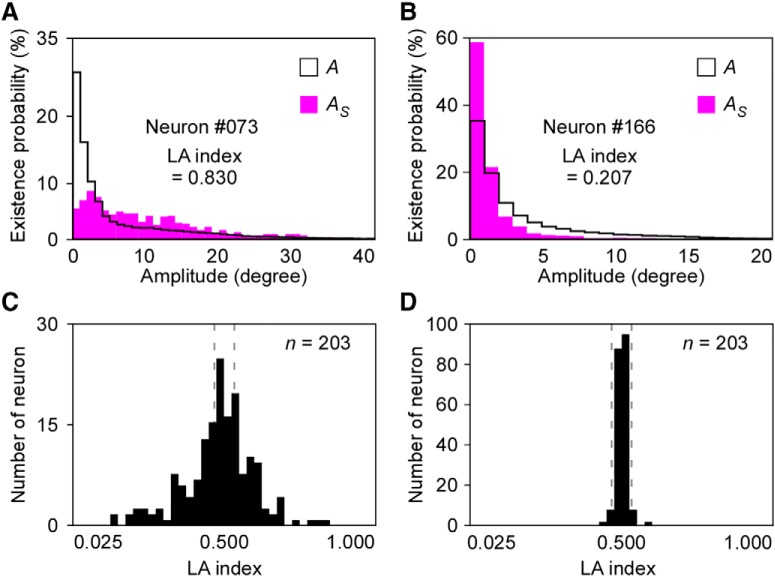
LA indexes of vM1 neurons and the shuffled data. ***A***, Representative example of a large LA index. Compared with *A*, *A_S_* was lower during small-amplitude whisking and higher during LA whisking in neuron #073. Neuron #073 tended to fire during LA whisking. ***B***, Representative example of a small LA index. ***C*,** The distribution of LA indexes for all recorded vM1 neurons. ***D***, Compared with the shuffled data, the LA indexes for recorded vM1 neurons showed significantly wider distribution (*p* < 0.001, *F* test). Dashed lines in ***C***, ***D*** indicate the range of mean ± 2 SD, which was calculated from the shuffled data.

A previous study reported that most vM1 neurons encode the amplitude of reciprocal vibrissa movement (Hill et al., 2011). In the present study, to examine the firing properties of vM1 neurons associated with whisk amplitude, we simultaneously recorded neuronal activity in the vM1 and vibrissa movement in head-fixed awake rats (Fig. 2*A*). Because whisking consists of rhythmic swinging along a longitudinal axis, we analyzed vibrissa movement by digitizing the vibrissal position as the azimuthal angle of the vibrissal shaft in horizontal plane images (Fee et al., 1997). Based on time-dependent changes in the vibrissal angle, we extracted the whisk amplitude using the wavelet transform and the Hilbert transform (Fig. 2*B*). We compared the distribution of the whisk amplitude during instances of neuronal firing (*A_S_*) with that throughout the total recording (*A*) for each vM1 neuron (Fig. 2*C*–*G*). In some neurons, the distribution of *A_S_* was biased toward a larger amplitude compared with that of *A*. These neurons were considered to fire preferentially during large-amplitude whisking (Fig. 3*A*). In contrast, other neurons increased firing when the whisking amplitude was small (Fig. 3*B*). To quantitatively evaluate the probability of neuronal firing that was correlated with whisk amplitude, we computed the Large-amplitude index (LA index) as a measure of the preference for large-whisk amplitude in each neuron (Fig. 2*G*). The LA index was defined as *P*(*A_S_* > *A’*), where *A’* is the median of *A*. The LA index ranged from 0, when the neuron fired only in a quiet state or during the whisk amplitudes that were smaller than the median, to 1, when the neuron fired only during the whisk amplitudes that were larger than the median.

To assess the distribution of the LA index, we compared the LA indexes of the recorded neurons with those of shuffled data (control data). The shuffled data were generated by randomly changing the interspike intervals of the recorded neurons while keeping the total numbers of spikes. The distribution of the LA index of all the recorded vM1 neurons (*n* = 203) and shuffled data are shown in Figure 3*C*,*D*, respectively. Compared with the shuffled data, the LA indexes for recorded vM1 neurons showed significantly wider distribution (*p* < 0.001 by *F* test). Dashed lines in Figure 3*C*,*D* indicate the range of mean ± 2 SD, which was calculated from the shuffled data. These contained neurons that tended to fire when the rats made large vibrissa movements (e.g., neuron #073; Fig. 3*A*) and neurons that were active during small-amplitude whisking as well as when the rats were in a quiet state (e.g., neuron #166; Fig. 3*B*). The LA index is thought to be closely related to corticofugal motor commands rather than to sensory information. Specifically, a previous study found a correlation between neuronal activity in the vM1 and whisk amplitude even after deprivation of the infraorbital nerve (Hill et al., 2011).

### LA index of PT and IT neurons

**Figure 4.**
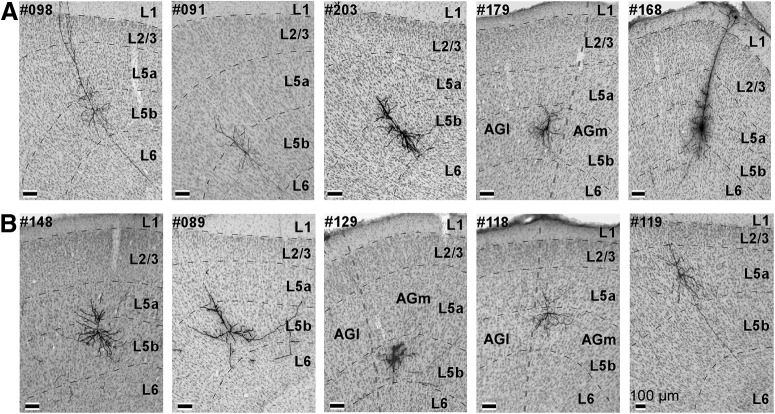
Cell bodies of the ten analyzed neurons. ***A***, Cell bodies of the PT neurons were visualized via the immunoperoxidase method. Cortical layers in coronal sections were identified via Nissl staining. All cell bodies of labeled vM1 neurons were localized in layer 5. ***B***, Cell bodies of the IT neurons. Thick dashed lines (#179, #129, #118) indicate the boundary between the AGm and AGl.

After completing the electrophysiological recordings, we injected plasmid, which expresses membrane-targeted fluorescent proteins, into the recorded neurons using the single-cell electroporation technique (Fig. 2*A*; Oyama et al., 2013). After tracing axonal projections from layer 5 of the vM1 at the single-neuron level, we obtained ten neurons (five PT and five IT neurons) that were acceptable for morphologic analysis (Figs. 1, 4). Most of the morphologically analized neurons (eight neurons) were found single in each brain, while only one brain contained two nicely labeled neurons. This is because the success rate of the electroporation in the present study was not so high. In the brain which contained the two labeled neurons, the axons derived from the two neurons did not overlap each other and thus we could segregate individual axons. The LA indexes of these ten neurons are shown in Tables 1, 2. The average of the LA indexes of the five PT neurons was 0.5903, and that of the LA indexes of the five IT neurons was 0.4183. When we sorted the analyzed neurons according to their LA indexes, we found that PT neurons exhibited larger indexes compared with IT neurons (*p* = 0.0472, Wilcoxon rank-sum test; Tables 1, 2). The mean firing rate of the PT neurons was 9.817 Hz and that of the IT neurons was 3.539 Hz. The difference in firing rates between the PT neurons and the IT neurons was not statistically significant (*p* = 0.0732, paired Student’s *t* test). Additionally, we did not find significant difference between PT neurons and IT neurons in firing properties associated with other motor variables such as midpoint of whisking, whisk frequency, and duration of protraction and retraction. Our results indicate that long corticofugal circuits to the brainstem were active during large-amplitude whisking. In contrast, corticocallosal neurons were active when the whisking amplitude was small or the vibrissae were at rest. Next, to explore the brain structures where the vM1 neurons sent motor information with respect to the LA index, we examined the axonal morphology of the recorded neurons.

### Axonal arborization of PT neurons

**Figure 5.**
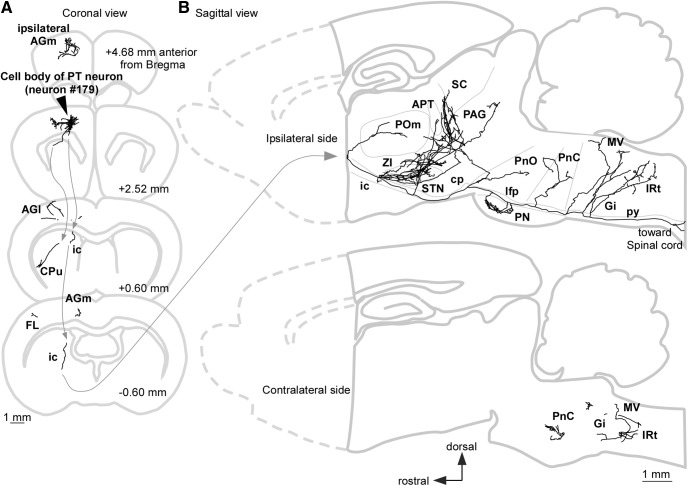
Reconstructed axonal arbor of a representative single PT neuron (#179; LA index was 0.5149), which exhibited a larger LA index than 0.5. ***A***, Projected axonal arborization to the coronal planes. This neuron sent its main axon to the spinal cord, and projected axon collaterals to the AGl, FL, and CPu on the ipsilateral side of the cell body. ***B***, Projected axonal arborization to the sagittal planes. Upper and lower drawings indicate axonal fibers on the ipsilateral and contralateral sides of the cell body, respectively. This neuron mainly projected to the ipsilateral subcortical areas. Some of the axon collaterals reached the contralateral lower brainstem. cp, cerebral peduncle; csc, commissure of the SC; ic, internal capsule; Gi, gigantocellular reticular nucleus; IRt, intermediate reticular nucleus; lfp, longitudinal fasciculus of the pons; MV, medial vestibular nucleus; PAG, periaqueductal gray; PnO/PnC, oral part and/or caudal part of the pontine reticular nucleus.

**Figure 6.**
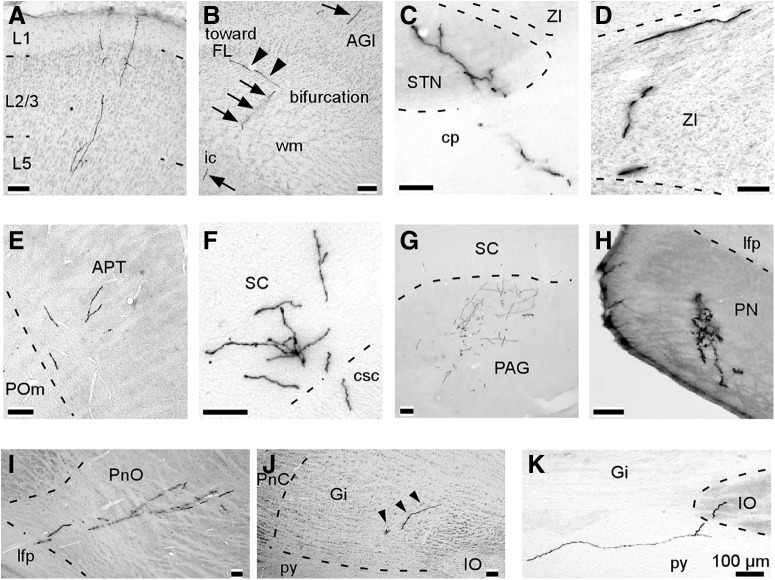
Representative examples of axonal collaterals in the target structures of PT neurons. These photographs were obtained from multiple PT neurons. ***A***, Axon collaterals in layers 1–5 of the ipsilateral Cg. ***B***, The main axon emitted a branch reaching the FL just before entering the ic. Arrows and arrowheads indicate the main axon and the branch, respectively. ***C***, ***D***, All of the PT neurons ipsilaterally projected to the STN and the ZI. ***E***, Most PT neurons sent axon collaterals to the APT. ***F***, ***G***, all neurons sent axon collaterals to the deep layers of the SC and PAG in the ipsilateral midbrain. ***H***, All of the PT neurons sent axon collaterals to the PN. ***I***, ***J***, most neurons innervated the ipsilateral PnO and Gi. Arrowheads indicate axonal collaterals. ***K***, Two neurons projected to the ipsilateral IO.

The PT neurons labeled in this study sent axon collaterals to the cerebral cortex, striatum, diencephalon, midbrain, pons, and medulla oblongata on the ipsilateral side of their cell bodies before their main axons terminated in the medulla oblongata or spinal cord, and some of their axon collaterals innervated the contralateral lower brainstem (Tables 1, 2). A representative PT neuron (#179) sent its main axon to the spinal cord, and collateral branches to the cerebral cortex [the AGm and forelimb area (FL)], caudate putamen (CPu; Fig. 5*A*,*B*), diencephalon [the posterior thalamic nucleus (POm), subthalamic nucleus (STN), and zona incerta (ZI)], midbrain [the anterior pretectal nucleus (APT), superior colliculus (SC), and periaqueductal gray], pons [the pontine nuclei (PNs) and pontine reticular nuclei], and medulla oblongata (the gigantocellular reticular nucleus, medial vestibular nucleus, and intermediate reticular nucleus) on the ipsilateral side of the cell body (Fig. 5*B*). Axon fibers that innervated the ipsilateral lower brainstem successively reached the pons (the caudal part of the pontine reticular nucleus) and the medulla oblongata (the gigantocellular reticular nucleus, medial vestibular nucleus, and intermediate reticular nucleus) on the contralateral side (Fig. 5*B*). In the cerebral cortex, most of the examined PT neurons (three of five neurons) expanded axons into the motor cortices adjacent to the cortex where the cell body existed (Fig. 6*A*), and two PT neurons sent axons to the ipsilateral FL (Fig. 6*B*). None of the PT neurons projected to the barrel field of the primary somatosensory cortex (S1BF). In the striatum and diencephalon, PT neurons very often projected ipsilaterally to the CPu (5/5), POm (3/5), STN (5/5), and ZI (5/5; Fig. 6*C*,*D*). Axon collaterals, which were derived from main axons in the internal capsule, entered the thalamus from the rostral side, and innervated the thalamic nuclei. Other collateral branches, which main axons emitted in the caudal side of the internal capsule and cerebral peduncle, reached the STN, ZI, and midbrain structures such as the APT, SC, and periaqueductal gray/mesencephalic reticular formation.

All five of the examined PT neurons sent axon collaterals to the ipsilateral brainstem, and some of them successively reached contralateral brainstem structures (Tables 1, 2). The majority of the PT neurons projected ipsilaterally to the APT (4/5; Fig. 6*E*), deep layers of the SC (5/5; Fig. 6*F*), and periaqueductal gray/mesencephalic reticular formation (5/5; Fig. 6*G*), and one neuron also projected to the contralateral midbrain. Axon collaterals, which were derived from main axons in the longitudinal fasciculus of the pons, always projected to the ipsilateral PN (5/5; Fig. 6*H*), and most of the collaterals successively innervated the contralateral PN (3/5). Axon collaterals of three PT neurons innervated the ipsilateral pontine reticular nucleus (Fig. 6*I*), and two of the three neurons reached the contralateral pontine reticular nucleus. PT neurons sent axon collaterals ipsilaterally to the gigantocellular reticular nucleus (4/5; Fig. 6*J*), inferior olive (IO; 2/5; Fig. 6*K*), and intermediate reticular nucleus (1/5), and almost half of them also contralaterally innervated the gigantocellular reticular nucleus (2/4), IO (1/2), and intermediate reticular nucleus (1/1).

Three PT neurons (#179, #203, and #098) sent main axons to the pyramidal decussation or the dorsal corticospinal tract, while the main axons of the other two neurons (#168 and #091) terminated in the medullary pyramidal tract. Since the PT neurons sent axon collaterals to brain structures before axon termination in the lower brainstem or spinal cord, these structures may share the same vibrissal motor information.

### Axonal arborization of IT neurons

**Figure 7.**
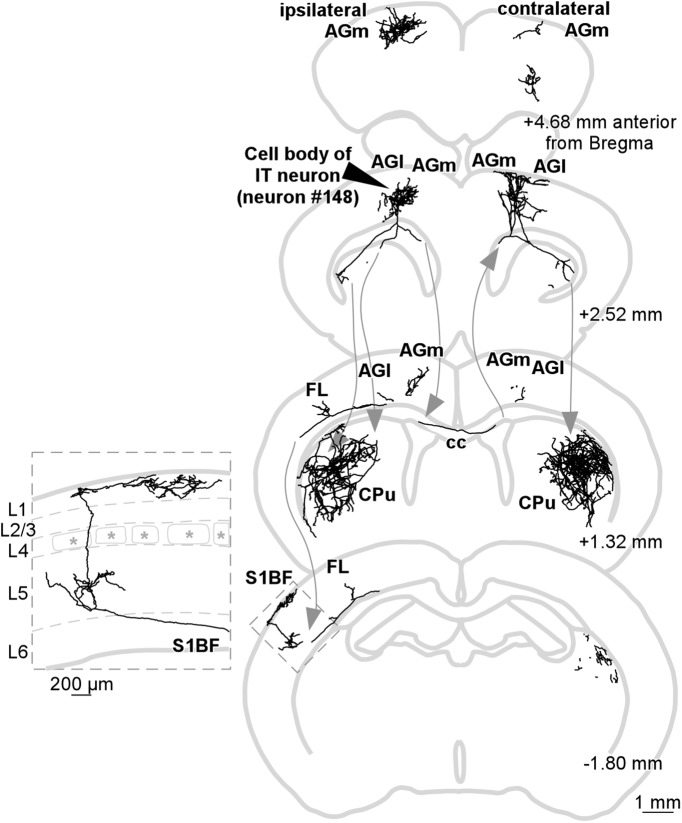
Reconstructed axonal arbor of a single IT neuron (#148; LA index was 0.2409), which exhibited a smaller LA index than 0.5. Axonal arborization of the barrel field of the S1BF (the rectangle) is magnified in the left-hand panel.

**Figure 8.**
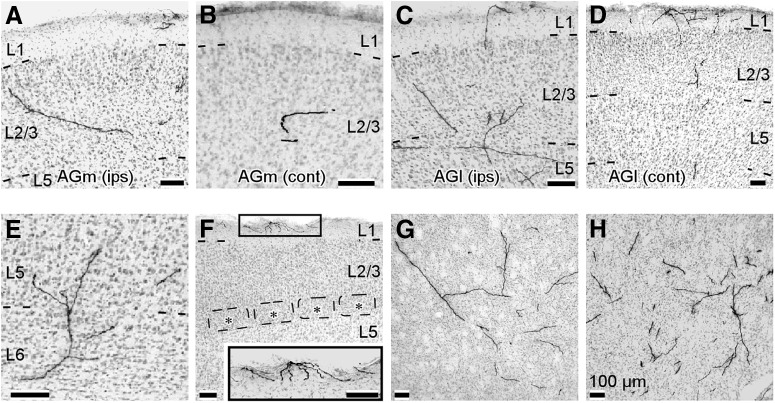
Representative examples of axon collaterals in the target structures of IT neurons. These photographs were obtained from multiple IT neurons. ***A***, ***B***, Axon fibers of IT neurons reached the upper layers (layers 1–2/3) of the ipsilateral AGm and contralateral AGm. ***C***, ***D***, IT neurons sent axons to layers 1–5 of the ipsilateral AGl and layers 1–5 of the contralateral AGl. ***E***, Axon fibers in layer 5–6 of the ipsilateral FL. ***F***, Two of the five IT neurons projected to layers 5–6 and layer 1 of the ipsilateral S1BF. Barrels are indicated by asterisks. The inset gives a magnified view of the rectangular area. ***G***, Three of the five IT neurons projected to the ipsilateral CPu. ***H***, All of the IT neurons innervated the contralateral CPu.

A representative IT neuron (#148) sent axons ipsilaterally to the AGm, AGl, S1BF, and CPu and contralaterally to the AGm, AGl, and CPu thorough the corpus callosum (Fig. 7). The majority of the IT neurons (four of five neurons) sent axons contralaterally thorough the corpus callosum to cerebral cortices (Fig. 8*A*–*D*) containing the cortical area symmetrical to the location of their cell bodies (Tables 1, 2). In two of the five IT neurons, collateral axons were emitted to the ipsilateral FL and S1BF (Fig. 8*E*,*F*). Axons of vM1 neurons, which project to these areas, are known to mostly innervate layer 1 and layers 5–6 (Veinante and Deschênes, 2003). In this study, axon fibers were projected to layer 1 and layers 5–6 of the S1BF (Fig. 8*F*), and to layers 5–6 of the FL. To summarize with respect to the cortical areas involved, IT neurons projected to the motor cortices related to vM1, FL, and S1BF. Three IT neurons projected to the ipsilateral CPu, and all of the IT neurons sent axons to the contralateral CPu (Fig. 8*G*,*H*). None of the IT neurons sent axons to any subcortical areas except the CPu.

### Dendritic morphology of PT and IT neurons

**Figure 9.**
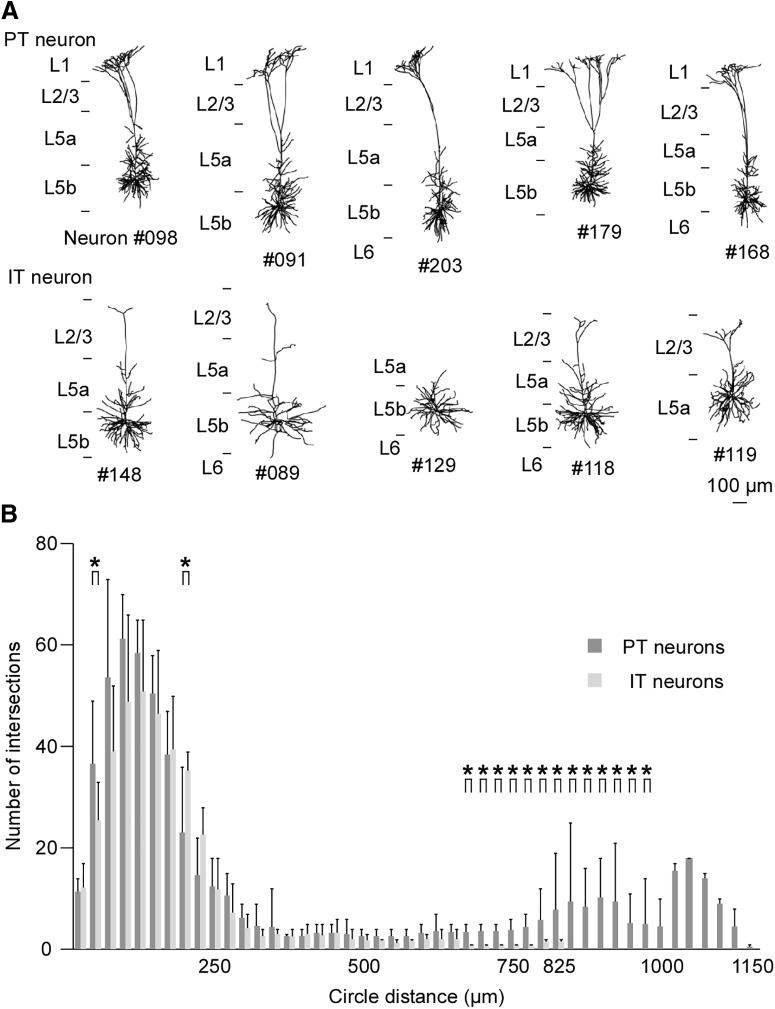
Dendrites and cell bodies of labeled PT and IT neurons. ***A***, Reconstruction of dendrites and cell bodies of PT neurons (upper row) and IT neurons (lower row). ***B***, The Sholl analysis revealed that PT neurons had more apical dendritic processes compared with IT neurons. Asterisks indicate statistically significant differences in the numbers of dendritic intersections between PT neurons and IT neurons (*p* < 0.05, Wilcoxon rank-sum test). The data are shown as mean and SD.

Figure 9*A* shows the reconstructed somatodendritic morphology of the labeled neurons in this study. Sholl analysis indicated that PT and IT neurons had different numbers of dendritic intersections in distances of 50, 200, and 675–975 µm from the cell bodies (*p* = 0.0476 at 50 µm, *p* = 0.0397 at 200 µm, *p* = 0.0317 at 675 µm, *p* = 0.0079 at 700 µm, *p* = 0.0079 at 725 µm, *p* = 0.0079 at 750 µm, *p* = 0.0079 at 775 µm, *p* = 0.0159 at 800 µm, *p* = 0.0159 at 825 µm, *p* = 0.0079 at 850 µm, *p* = 0.0079 at 875 µm, *p* = 0.0079 at 900 µm, *p* = 0.0079 at 925 µm, *p* = 0.0079 at 950 µm, *p* = 0.0476 at 975 µm, Wilcoxon rank-sum test; Fig. 9*B*). The difference in the number of intersections in a distance of 675–975 µm from the cell bodies is likely attributable to the richer apical tuft of PT neurons (Fig. 9*A*). PT neurons in layer 5 of the motor cortex are known to have longer apical dendrites and prominent terminal arbors in layer 1, and apical dendrites of IT neurons terminate in the middle layers (Veinante and Deschênes, 2003; Kim et al., 2015). Therefore, our result was in agreement with previous reports. The rostromedial portion of the ventral anterior-ventral lateral motor thalamic nuclei (VA-VL) projects to layer 1 of the motor cortex, and the caudolateral portion of the VA-VL innervates the middle layers of the motor cortex (Kuramoto et al., 2009). The basal ganglia send GABAergic efferent projections to the rostromedial portion of the VA-VL, while glutamatergic input from the cerebellum enters the caudolateral portion (Kuramoto et al., 2009). Dendritic morphology indicated that the information from the basal ganglia may be sent not to IT neurons but to PT neurons. The differences in firing properties between PT and IT neurons are thought to relate to differences in their dendritic inputs.

## Discussion

In the present study, we demonstrated that PT neurons in layer 5 of the vM1 preferentially fired when rats made large-amplitude whisking, while IT neurons showed high activity during small or null whisking. Furthermore, we described detailed projection patterns of descending axons derived from these two groups of layer 5 neurons at the individual neuron level. We used the single neuron labeling technique combined with the simultaneous recording of vibrissal movement and single neuron firing in awake rats to demonstrate, for the first time, a direct correspondence between firing property and morphologic classification in layer 5 neurons in the cortex according to axonal distribution (Shepherd, 2013; Harris and Shepherd, 2015). If the two different corticofugal circuits derived from the motor cortex encode motor information differently, the present findings provide a basis for understanding of mechanisms of motor control and motor-associated information processing. In the previous studies where they analyzed correlations between firing properties of neurons and anatomic data such as location of cell bodies or mRNA expressions (Isomura et al., 2009; Deschênes et al., 2016), juxtacellular recording and labeling technique that enables researchers to visualize morphology of single neurons electrophysiologically identified by unit recordings (Pinault, 1996) was used. On the other hand, it is actually difficult to clearly label long projecting axons by the conventional juxtacellular labeling technique, because dye injected into a cell body is considered to be delivered to axons by diffusion and the concentration of the dye in the distal part of the axons is low. The gene transfer of plasmids encoding fluorescent proteins by the single-neuron electroporation increase labeling intensity and thus enable the more precise analysis of the axonal arborization at a single-neuron level.

Some PT neurons have been found to send axons to the whisking CPG in the lower brainstem (Moore et al., 2013; Takatoh et al., 2013; Kita et al., 2014). The excitatory input from PT neurons has the potential to activate the FN through the CPG when the whisking amplitude is large. Our results suggest that the principal role of PT neurons is to induce large-amplitude whisking through the CPG and FN. PT neurons sent axons not only to the CPG but also to many other subcortical regions. The pons and medulla oblongata received abundant axon collaterals from long descending PT neuron projections, and most PT neurons projected to the PN and IO. Since these nuclei relay information to the cerebellum, copies of motor commands are expected to influence cerebellar functions, such as the coordination, precision, and accurate timing of movements (Wolf et al., 2009). In addition, all of the PT neurons in our study projected ipsilaterally not only to the CPu but also to the STN and ZI. These regions are known to have strong connections with the basal ganglia (Kita et al., 2014), which is associated with voluntary motor movement, learning, cognition, and emotion (Stocco et al., 2010). As previously reported (Deschênes et al., 1994; Alloway et al., 2009; Kita and Kita, 2012), most of the five PT neurons also sent axons to the POm, APT, and SC. The APT and ZI are known to innervate the POm, and have a strong inhibitory influence on the thalamus (Giber et al., 2008). The SC has many functions including control of vibrissa movement (Hemelt and Keller, 2008), vibrissal sensory processing (Bezdudnaya and Castro-Alamancos, 2014), and locomotion (Felsen and Mainen, 2008). Whisking motor commands may be integrated with such information in the SC, and thus contribute to exploring activity. Overall, our data and that from previous studies indicate that copies of cortical motor commands given by PT neurons are shared with the cerebellum, basal ganglia, and SC, and that such data contribute to motor-associated functions including fine movement and motor control.

The decrease in IT neuronal firing rate during large-amplitude whisking indicates that the motor information given by IT neurons might differ from the motor commands that directly move the vibrissae. Given that the firing properties and axonal projections of IT neurons are different from those of PT neurons, IT neurons likely possess different roles in motor function.

Gao et al. (2003) reported that unilateral lesion of the motor cortex caused changes of whisking amplitude and velocity not only on the contralateral side but also on the ipsilateral side that is mainly modulated by the contralateral motor cortex (Gao et al., 2003). This suggests that the activity of the contralateral cortex was affected by the elimination of cortico-callosal axons from the lesioned cortex. In awake animals, a neuron group in layer 2/3 of the frontal cortex is known to exert axonal projection to the contralateral cortex (Kawaguchi, 2017) and has been reported to reduce firing rate of the target area during whisking (Sreenivasan et al., 2016). Because one of the most striking characteristics of axonal morphology in IT neurons is the cortico-callosal projection (Figs. 7, 8*A*–*D*; Shepherd, 2013) similarly with the group of layer 2/3 neurons, it is speculated that IT neurons cooperate with the cortico-callosal neurons in layer 2/3 to associate the right and left cortex and to contribute to the coordinated movement of bilateral vibrissae.

The CPu, which is a major component of the basal ganglia (Gerfen, 1992), was an important target for the IT neurons examined in this study. Because most of the IT neurons projected bilaterally to the CPu, IT neurons might affect bilateral basal ganglia activities. Additionally, the present study shows that two IT neurons sent axons to the ipsilateral S1BF, while no PT neurons projected to the S1BF, in agreement with the previous report where most of motor cortex neurons projecting to the S1BF were IT neurons (Veinante and Deschênes, 2003). S1BF neurons are thought to receive not only tactile signals but also other inputs including those associated with decision-making (O'Connor et al., 2010) and vibrissal motor information (Hill et al., 2011). Our results suggest that vibrissal motor information processed by IT neurons is sent directly to S1BF, and thus that this information is used for sensorimotor integration.

## Synthesis

Reviewing Editor: Muriel Thoby-Brisson, CNRS UMR 5287 Université Bordeaux Decisions are customarily a result of the Reviewing Editor and the peer reviewers coming together and discussing their recommendations until a consensus is reached. When revisions are invited, a fact-based synthesis statement explaining their decision and outlining what is needed to prepare a revision will be listed below. The following reviewer(s) agreed to reveal their identity: Nathan Cramer, Nicole Mercer Lindsay.

Below are the reviewers' comments:

In the manuscript “Projection patterns of corticofugal neurons associated with vibrissa movement”, the authors examine the relationship between projection patterns of pyramidal neurons in layer 5 of the primary motor cortex and their corresponding spiking activity during whisking in restrained, but awake rats. The research seeks to address how higher brain centers, such as the motor cortex, contribute to motor behaviors. The authors use a combination of electrophysiology, video monitoring and histological techniques to determine which types of projecting neurons regulate aspects of whisking behaviors. Overall the manuscript shows that layer 5 neurons in vM1 that project to the brainstem fire preferentially during larger amplitude whisker movements while layer 5 neurons in vM1 that project only to the other cortical areas and the basal ganglia fire preferentially during small amplitude whisking. This result is the first to clearly show two distinct pathways have different firing preferences related to amplitude of movement. This manuscript also adds beautiful full reconstructions of pyramidal neurons of layer 5 vM1 to a small pool of other such reconstructions. These reconstructions, in addition to definitively showing pyramidal tract neurons vs intratelencephalic neurons, confirm the broad collateralization patterns seen in other such reconstructions.

The experiments rationally designed and the results clearly explained. A few points of clarification would add to its impact.

1. Is it correct that microstimulation was not performed in rats from which extracellular recordings were obtained? As the authors point out, the location of M1 is not widely contested so this is more for clarity.

2. The statistics section reports that 203 neurons were recorded which works out to essentially 5 cells per rat. How many of these cells per rat were labeled juxtacellularly? Given how the brain was sectioned into two different dimensions, it would seem almost impossible to accurately follow projections from a single cell after multiple cells were labeled.

3. Were the 10 cells used in the reconstruction analysis the only cells attempted to be labeled in those brains (i.e. one brain, one labeling attempt)? Even if only one cell seemed to be successfully labeled per brain, I don't think one could reliably say all of the labeled processes came from that one cell if multiple labeling attempts were made.

4. I don't think the statement starting at line 236 which tries to interpret the overall proportion of IT and PT that are related to small or large amplitude whisking based on a subset of 10 cells is valid. An N of 10 is too small for this conclusion.

5. It would be nice to see the boundary between the AGm and the AGl in Figure 3 in particular but also in the reconstructions and images. The authors have the nissl stain so it would be very useful to see how the vM1 as determined by stimulation compares with the cytoarchitecture. As is, it felt vague connected.

6. When describing the ICMS the authors don't describe whether the vibrissa movements were protractive or retractive. If a record of this was kept, it would be useful to see how similar the vM1 division is between this study and previous studies.

## Author Response

Dear Sirs,

Hearty thanks for reviewing our manuscript. We are returning herewith a manuscript revised according to reviewer's comments.

1. Is it correct that microstimulation was not performed in rats from which extracellular recordings were obtained? As the authors point out, the location of M1 is not widely contested so this is more for clarity.

Answer to the reviewer comment #1:

We did not performed intracortical microstimulation (ICMS) in the rats from which unit recordings were obtained because we were afraid that the electrode penetration of ICMS would give influences to the region of interest and make it difficult to obtain recordings. To carefully test the functional map of the motor cortex for vibrissa movement, we additionally performed ICMS with another stimulus protocol (bipolar current pulses, Haiss and Schwartz, 2005). The results of ICMS are summarized in the new Figure 1. The additional experiment shows the roughly the same results with that of Haiss and Schwartz (2005), where ICMS in the small area near the bregma induced vibrissal protractions, while retractions were evoked by ICMS in the larger cortical area lateral to the protraction area.

2. The statistics section reports that 203 neurons were recorded which works out to essentially 5 cells per rat. How many of these cells per rat were labeled juxtacellularly? Given how the brain was sectioned into two different dimensions, it would seem almost impossible to accurately follow projections from a single cell after multiple cells were labeled.

Answer to the reviewer comment #2:

We applied the single-cell electroporation procedure to 3-4 recorded neurons in each brain (2 neurons with GFP and other 2 neurons with mRFP). However, most of the labeled neurons (8 neurons) were found single in each brain because the success rate of the electroporation in the present study was not so high. Only one brain contained 2 labeled neurons. In this case, we could segregate individual axons because the axons derived from the 2 neurons did not overlap each other. We added an explanation in Results section of the main texts (page 6, lines 228-232).

3. Were the 10 cells used in the reconstruction analysis the only cells attempted to be labeled in those brains (i.e. one brain, one labeling attempt)? Even if only one cell seemed to be successfully labeled per brain, I don't think one could reliably say all of the labeled processes came from that one cell if multiple labeling attempts were made.

Answer to the reviewer comment #3:

As described above, we applied the plasmid injection to 3-4 recorded neurons in each brain. We performed plasmid injections with enough distances between places of the injections so that we could identify labeled neurons and correlate them with their recordings. We also used two kinds of reporter proteins to increase the chance of injection trial (2 neurons with GFP, other 2 neurons with mRFP). We added an explanation for the places of the injections in the Materials and Methods section (page 3, lines 122-124).

4. I don't think the statement starting at line 236 which tries to interpret the overall proportion of IT and PT that are related to small or large amplitude whisking based on a subset of 10 cells is valid. An N of 10 is too small for this conclusion.

Answer to the reviewer comment #4:

We agree to this opinion. We removed the description (page 6, lines 244-247) from Results section.

5. It would be nice to see the boundary between the AGm and the AGl in Figure 3 in particular but also in the reconstructions and images. The authors have the nissl stain so it would be very useful to see how the vM1 as determined by stimulation compares with the cytoarchitecture. As is, it felt vague connected.

Answer to the reviewer comment #5:

We put a line showing the boundary between the AGm and AGl in Figure 3A. The boundary is not parallel to the vertical axis actually and the line indicates the boundary at layer 5 level. We also illustrate the boundary in the photographs of cell bodies (new Figure 4, #118, #129, #179).

6. When describing the ICMS the authors don't describe whether the vibrissa movements were protractive or retractive. If a record of this was kept, it would be useful to see how similar the vM1 division is between this study and previous studies.

Answer to the reviewer comment #6:

We additionally performed ICMS with another stimulus protocol (bipolar current pulses, Haiss and Schwartz, 2005). The results of ICMS are summarized in the new Figure 1. The additional experiment shows the roughly the same results with that of Haiss and Schwartz (2005), where ICMS in the small area near the bregma induced vibrissal protractions, while retractions were evoked by ICMS in the larger cortical area lateral to the protraction area.

We hope that revised manuscript meets your approval and will be more suitable for publication in your journal.
